# Conserved immunomodulatory transcriptional networks underlie antipsychotic-induced weight gain

**DOI:** 10.1038/s41398-021-01528-y

**Published:** 2021-07-22

**Authors:** Rizaldy C. Zapata, Besma S. Chaudry, Mariela Lopez Valencia, Dinghong Zhang, Scott A. Ochsner, Neil J. McKenna, Olivia Osborn

**Affiliations:** 1 Division of Endocrinology and Metabolism, School of Medicine, University of California San Diego, La Jolla, CA 92093 USA; 2 Signaling Pathways Project and Department of Molecular and Cellular Biology, Baylor College of Medicine, Houston, TX 77030 USA

**Keywords:** Schizophrenia, Predictive markers, Molecular neuroscience

## Abstract

Although antipsychotics, such as olanzapine, are effective in the management of psychiatric conditions, some patients experience excessive antipsychotic-induced weight gain (AIWG). To illuminate pathways underlying AIWG, we compared baseline blood gene expression profiles in two cohorts of mice that were either prone (AIWG-P) or resistant (AIWG-R) to weight gain in response to olanzapine treatment for two weeks. We found that transcripts elevated in AIWG-P mice relative to AIWG-R are enriched for high-confidence transcriptional targets of numerous inflammatory and immunomodulatory signaling nodes. Moreover, these nodes are themselves enriched for genes whose disruption in mice is associated with reduced body fat mass and slow postnatal weight gain. In addition, we identified gene expression profiles in common between our mouse AIWG-P gene set and an existing human AIWG-P gene set whose regulation by immunomodulatory transcription factors is highly conserved between species. Finally, we identified striking convergence between mouse AIWG-P transcriptional regulatory networks and those associated with body weight and body mass index in humans. We propose that immunomodulatory transcriptional networks drive AIWG, and that these networks have broader conserved roles in whole body-metabolism.

## Introduction

Antipsychotic drugs are effective medications for the treatment of psychiatric disease but have significant side effects, including antipsychotic-induced weight gain (AIWG) [1, 2]. AIWG has been shown to increase the risk of both developing metabolic syndrome [3] and of mortality [4]. Approximately 20% of patients treated with a broad range of APs gain clinically significant amounts of weight (>7% of their baseline weight) [1]. Notably, the incidence of diabetes among second-generation antipsychotic users is four times higher than age-matched, race-matched, and sex-matched controls [5]. Metabolic side effects are also the most commonly reported reason for noncompliance with second generation antipsychotic medications [6]. Although almost all antipsychotics result in some degree of weight gain [1], the extent of weight gain varies between individuals [7, 8]. Some individuals can gain a pound per week of treatment, while others are relatively refractory to weight gain [1, 9–13].

Olanzapine is one of the most clinically effective antipsychotic drugs but also results in highly significant weight gain in many patients [1, 2, 9, 13, 14]. Therefore, there is a need to determine which patients are less susceptible to the metabolic side effects of antipsychotics and would be good candidates for drugs such as olanzapine, and conversely, identifying patients that should be prescribed alternative antipsychotics with less weight gain liabilities. Although olanzapine is known to target multiple receptors including serotonergic (5-HT_2_ and 5-HT_6_), dopaminergic (D2, D3, and D4), muscarinic (M1–5), α_1_ adrenergic and histaminergic H1 receptors, the underlying biological mechanisms responsible for AIWG are incompletely understood. Previous studies have shown that the effect of olanzapine on AIWG can be effectively modeled in mice [15, 16]. Here, we used gene expression profiling in a mouse model to illuminate signaling pathways predisposing to AIWG, and to identify genes with potential as blood-based biomarkers of AIWG in humans.

## Materials and methods

### Mouse studies

#### Antipsychotic-induced food intake and weight gain study

Forty female C57BL/6J mice (stock #000664) were purchased from Jackson Laboratory at nine weeks of age. After seven days of acclimation to experimental conditions (12:12 light–dark, 20–21 °C, 50% humidity), blood was collected and total RNA isolated from blood using Mouse RiboPure Blood RNA Isolation Kit (AM1951, Invitrogen, Carlsbad, CA, USA) according to the manufacturer’s instructions. All mice were transitioned to a 45% high-fat diet (HFD) compounded with 54 mg/kg olanzapine (Research Diets, Inc., D09092903) for 14 days. The olanzapine dose selected results in mouse plasma levels (21 ± 5 ng/mL) that are similar to the levels observed in humans treated with olanzapine (10–50 ng/mL) [17]. All 40 mice were singly housed throughout the study, and food intake was measured daily, and body weight was measured every other day for 14 days. At the end of the study, mice were anesthetized and sacrificed. Hypothalami were dissected and stored at −80 °C for qPCR analysis. Food intake was analyzed for all 40 mice. We then classified the top five weight gainers as weight gain-“prone” mice (AIWG-P) and the five least weight gainers as weight gain “resistant” mice (AIWG-R). Sample sizes were selected based on previous studies [15]. No randomization or blinding was applied in these studies. All experiments were approved by and conducted in accordance with the University of California, San Diego IACUC.

#### High-fat diet-induced weight gain study

Baseline blood was collected from C57BL6 mice and total RNA isolated using Mouse RiboPure Blood RNA Isolation Kit (AM1951, Invitrogen, Carlsbad, CA, USA) according to the manufacturer’s instructions. Starting body weight was recorded before mice were transitioned from normal chow to a 45% high-fat diet (HFD) for 14 days, at which point body weight was recorded and body weight gain calculated. Mice were then divided into high-fat diet-induced weight gain-prone (HFWG-prone) and HFWG-resistant groups.

### RNA sequencing

The RNA extracted from blood was sequenced at the UCSD Institute for Genomic Medicine. The quality of the RNA was assessed using the Tapestation 2200 (Agilent) and Libraries prepared using TruSeq Library prep kits (Illumina), and then run on the Tapestation high-sensitivity DNA assay kits to ensure the correct library size. Libraries were quantified using the Qubit^®^ 2.0 Fluorometer, pooled, and run on the Illumina NovaSeq 6000 (Illumina). Reads were mapped to the mouse transcriptome using Bowtie2 algorithm [18] and counted as reads per gene using RSEM [19] and then analyzed using the statistical algorithm limma (RRID:SCR_013027). RNA-sequencing data have been archived in the Gene Expression Omnibus (GEO) database GSE157438 (RRID:SCR_005012). Human RNA-seq data were obtained from a previous published study where blood samples were sequenced from patients before three months of treatment with antipsychotics [20]. This study conformed to the international standards for research ethics and was approved by the Cantabria Ethics Institutional Review Board (IRB).

### Hypothalamus RNA isolation and RT-qPCR

Total RNA was isolated using Trizol (Invitrogen, Carlsbad, USA) and purified using RNAeasy (Qiagen) according to the manufacturer’s protocol. RNA concentration and quality were measured on a NanoDrop (NanoDrop Technologies, Rockland, DE, USA). cDNA was prepared and qPCR was performed with Perfecta FastMix (95073-05 K, VWR) and specific primers (Supplementary file section 1). Gene expression was normalized using Pgk1 and Hprt as the housekeeping genes using the ΔΔCt method. Data were analyzed by Student’s *t*-test. Normal distribution was tested using Shapiro-Wilk test prior to proceeding with a Student’s *t*-test with Welsh’s correction, thus not assuming equal variances between populations. *p* < 0.05 was considered statistically significant using Graphpad Prism.

### PANTHER Gene Ontology analysis

PANTHER Gene Ontology analysis of gene sets was carried out using the Panther Overrepresentation Test (Release 20200728; [21]) with the following parameters: Reference List, Mus musculus (all genes in the database); Test Type, FISHER; Correction, FDR.

### Signaling Pathways Project transcriptional regulatory network analysis

Transcriptional regulatory network analysis of gene sets was carried out as previously described [22, 23]. Consensomes are gene lists ranked according to measures of the strength of their regulatory relationship with upstream signaling pathway nodes derived from independent publicly archived transcriptomic or ChIP-Seq datasets [20]. To generate ChIP-Seq consensomes, we first retrieved processed gene lists from ChIP-Atlas [24], in which genes are ranked based on their mean MACS2 peak strength across available archived ChIP-Seq datasets in which a given pathway node is the IP antigen. Of the three stringency levels available (10, 5, and 1 kb from the transcription start site), we selected 5 kb. We then mapped the IP antigen to its pathway node category, class, and family, and organized the ranked lists into percentiles to generate the node ChIP-Seq consensomes [22]. Genes in the 95th percentile of a given node consensome were designated high-confidence transcriptional targets (HCTs) for that node and used as the input for the HCT intersection analysis using the Bioconductor GeneOverlap analysis package implemented in R as previously described [21]. *p*-values were adjusted for multiple testing by using the method of Benjamini and Hochberg to control the false discovery rate as implemented with the p.adjust function in R, to generate *q*-values. Evidence for a transcriptional regulatory relationship between a node and a gene set was represented by a larger intersection between the gene set and HCTs for a given node than would be expected by chance after FDR correction (*q* < 0.05).

### Other statistical analyses

Genes mapping to the Mammalian Phenotype Ontology phenotypes “decreased total body fat mass” or “slow postnatal weight gain” were retrieved from the Monarch [43], IMPC [44], or MMPC [45] resources. A hypergeometric analysis (Graphpad, Prism 7.0) was used to estimate the overrepresentation of genes mapped to these phenotypes among nodes with HCT/AIWG-P intersections of *q* < 0.05. There was enrichment of mouse orthologs of genes whose expression correlated with weight gain and body mass index in humans. Hypergeometric analysis was used to analyze overrepresentation of genes in the E2 ubiquitin-conjugating and E3 ubiquitin ligase enzyme classes among genes at the intersection of the AIWG-P gene set and mouse orthologs of the HWG/BMI gene set. The universe was set at the total number of genes currently annotated in the SPP database (24703).

## Results

### Olanzapine treatment results in variation in weight gain in mice

**Fig. 1:**
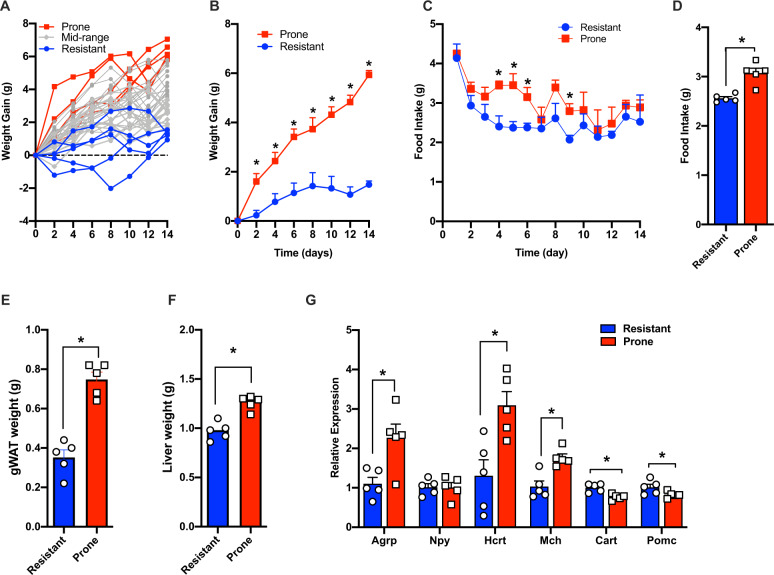
Weight gain and food intake in response to olanzapine treatment. **A**. Individual daily weight gain in all 40 mice. **B** Average weight gain. **C** Average daily food intake. **D** Average food intake across 14 days of treatment. **E** gWAT. **F** Liver weight. **G** Hypothalamic gene expression in top five most weight gain ‘prone’ mice and five most ‘resistant’ to weight gain. *p < 0.05 determined by students *t*-test and corrected with 2-stage set-up method of Banjamini Krieger and Yekutieli for multiple comparisons with FDR = 0.10. Data are expressed as mean ± standard error.

Although all mice gained weight in response to olanzapine/HFD feeding, the extent of weight gain varied from ~1 g to ~7 g over the course of 14 days of treatment (Fig. 1A). We identified the five mice that gained the most weight (‘prone’, AIWG-P) and compared them with five mice at the other end of the spectrum that were ‘resistant’ to weight gain (AIWG-R). The average weight gain of the AIWG-P group was 6.3 g compared with 1.3 g in the AIWG-R group (Fig. 1B). Throughout the study, the AIWG-P mice ate approximately 0.5 g (19% more food) per day than the AIWG-R group (average ‘AIWG-P’ = 3.1 g/day vs ‘AIWG-R’ = 2.6 g/day) (Fig. 1C, D). After 14 days of treatment, the mice were sacrificed, and tissues dissected. As expected, AIWG-P mice had significantly higher gonadal white adipose fat mass (gWAT) (Fig. 1E) and greater liver mass (Fig. 1F) than the AIWG-R group.

Given that olanzapine is known to induce orexigenic gene expression in the hypothalamus [15], we next compared hypothalamic expression of appetite-regulating genes in the AIWG-P and AIWG-R groups. Hypothalamic expression of *Agrp* (agouti-related neuropeptide), *Hcrt* (hypocretin neuropeptide precursor), and *Mch* (melanin-concentrating hormone) was strongly elevated in the AIWG-P cohort relative to the AIWG-R cohort. In contrast, expression of the anorexigenic genes *Pomc* (proopiomelanocortin) and *Cart* (cocaine and amphetamine-related transcript) was lower in the AIWG-P relative to the AIWG-R cohort (Fig. 1G).

**Fig. 2:**
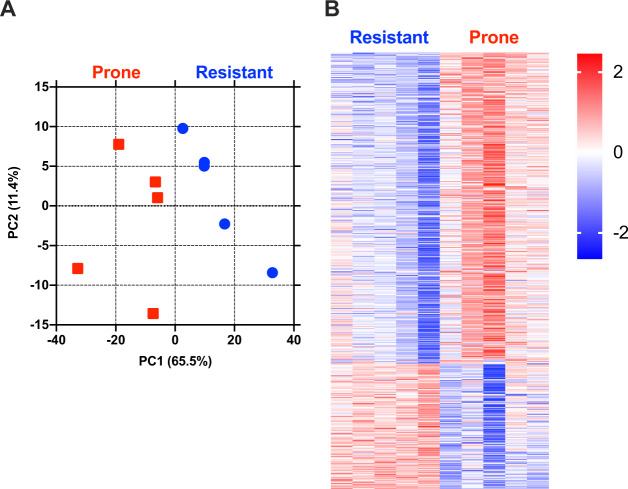
RNA sequencing analysis displays differential gene expression between ‘prone’ and ‘resistant’ cohorts. **A**. Principal component analysis. **B** Heat map of differentially expressed genes between cohorts.

**Fig. 3:**
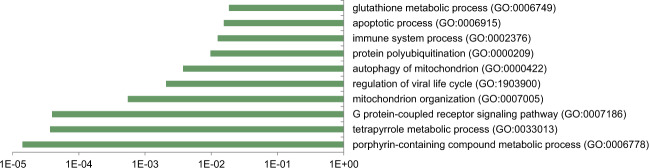
PANTHER Gene Ontology analysis of the AIWG-P gene set. Shown are terms with *q* < 0.05; full numerical data are provided in Supplementary file section 3. No significantly overrepresentation terms were identified in the AIWG-R gene set.

We next prepared RNA from blood taken at baseline from individuals subsequently assigned to the AIWG-P and AIWG-R cohorts and conducted RNA sequencing (Fig. 2A). We identified 558 genes that were significantly differentially expressed (FC > ± 1.25, *p* < 0.05) at baseline between the AIWG-P and AIWG-R cohorts (Fig. 2B and Supplementary file section 2), of which 389 were elevated in AIWG-P > AIWG-R (AIWG-P gene set), and 169 were elevated in AIWG-R > AIWG-P (AIWG-R gene set). Panther Gene Ontology (GO) analysis [25] of the AIWG-P gene set indicated enrichment of genes mapping to a number of biological processes with known relevance to lipid metabolism (Fig. 3; Supplementary file section 3). For example, the overlapping porphyrin (GO:0006778; *q* = 8e-8) and tetrapyrole (GO:0033013; *q* = 3e-5) metabolic process pathways encompass genes encoding enzymes with characterized roles in energy metabolism, including *Hmox1* [26, 27], *Alas1*, and *Alad* [28]. Similarly, mitochondrial autophagy (GO:0000422; *q* < 4e-3) is a fundamental process in cellular energy metabolism that has been linked to a spectrum of signature disorders of the metabolic syndrome, including type 2 diabetes and obesity [29–32]. Of particular interest given the well-characterized connection between immunity, inflammation and obesity [33, 34] was the enrichment for the immune system (GO:0002376; *q* < 1e-2) process (Fig. 3).

### AIWG transcriptional regulatory networks are enriched for immunomodulatory and inflammatory signaling nodes

Although the Panther GO analysis illuminated processes in which AIWG-P genes were functionally involved, it was much less informative on the extent to which genes in the AIWG-P and AIWG-R sets had common upstream transcriptional regulators. The Signaling Pathways Project (SPP) curates archived ‘omics’ datasets to compute consensus-regulatory signatures, or consensomes, which rank genes based on measures of the strength of their transcriptional regulatory relationships with specific upstream signaling pathway nodes [22]. As such, SPP represents a reduced-bias environment within which to identify high-confidence transcriptional targets (HCTs) of these nodes. We recently described HCT intersection analysis, in which we computed coronavirus infection HCTs against human signaling node HCTs to identify candidate host pathways mediating the transcriptional response to CoV infection [23]. To gain insight into signaling pathway nodes whose gain or loss of transcriptional function contributes to AIWG, we next applied HCT intersection analysis to compute intersections between the AIWG-P and AIWG-R gene sets and HCTs for mouse signaling pathway nodes. We interpreted the extent and significance of these intersections as evidence for loss or gain of function of signaling nodes in weight gain proneness or resistance.

**Fig. 4:**
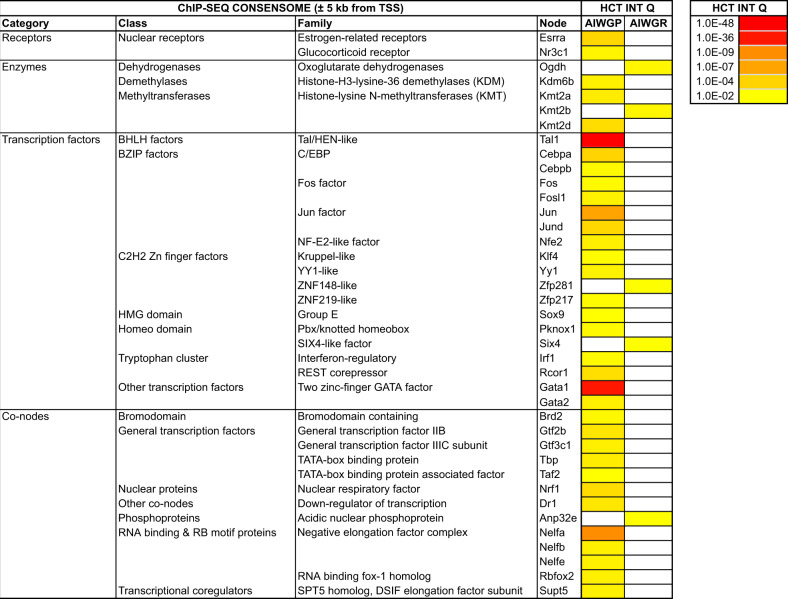
ChIP-Seq high-confidence transcriptional target (HCT) intersection analysis of the AIWG-P and AIWG-R gene sets and mouse signaling nodes. *q*-values were generated using the GeneOverlap analysis package in R as previously described (Ochsner et al. 2020) [23]. White cells represent *q* > 5e-2 intersections. Shown are *q* < 0.01 intersections; full numerical data are provided in Supplementary file section 4. The universe for the intersection was set at a conservative estimate of the total number of transcribed genes in the mouse genome (30,000; [71]).

Figure 4 shows node HCT intersections (*q* < 0.01) with the AIWG-P and AIWG-R gene sets; the full set of intersections is provided in Supplementary file section 4. All *q*-values cited in the following section were generated using the R GeneOverlap package as previously described [23]. Strikingly, node HCT intersections were strictly partitioned between the AIWG-P and AIWG-R gene sets such that no nodes had HCT intersections with both gene sets. Consistent with the Panther GO analysis (Fig. 3), and the well-documented evidence connecting inflammation, the immune system, and metabolic disease [29, 31, 32, 35], we observed HCT intersections for numerous immunomodulatory and inflammatory nodes that were specific to the AIWG-P gene set. The two most robust AIWG-P intersections were for Tal1/SCL (2e–48) and Gata1 (1e–36), which play critical roles in the development of hematopoietic lineages with fundamental roles in immune processes [36, 37]. Other prominent immunomodulatory nodes with AIWG-P intersections included members of the AP-1 (21339212; Jun, 4e-7; Jund, 4e-4; Fos, 7e-3), interferon-regulatory ([38]; Irf1, 7e-3; Irf9, 2e-2; Irf2, 4.9e-2), STAT ([38]; Stat2, 2e-2; Stat3, 3e-2; Stat1, 3e-2), C/EBP ([39]; Cebpa, 3e-4; Cebpb, 6e-3; Cebpd; 2e-2), and SMAD ([40]; Smad1, 3e-2) transcription factor families. Given the wealth of studies connecting circadian rhythms and metabolic dysfunction, and our recent observations of the importance of circadian rhythms in AIWG [41], we were interested to note significant intersections of the AIWG-P gene set with HCTs of several transcription factors with characterized roles in circadian biology, including Nr3c1/GR (*q* = 5e-3; [42]), MAX (4e-2 [43]), and members of the estrogen-related receptor (Esrra, *q* = 4e-4, Esrrb, 2.4e-2 [44]) and C/EBP (Cebpa, 3e-4; Cebpb, 6e-3; Cebpd, 2e-2; [45]) families.

### Candidate drivers of AIWG transcriptional programs are enriched for genes supporting body fat mass and weight gain in mice

**Fig. 5:**
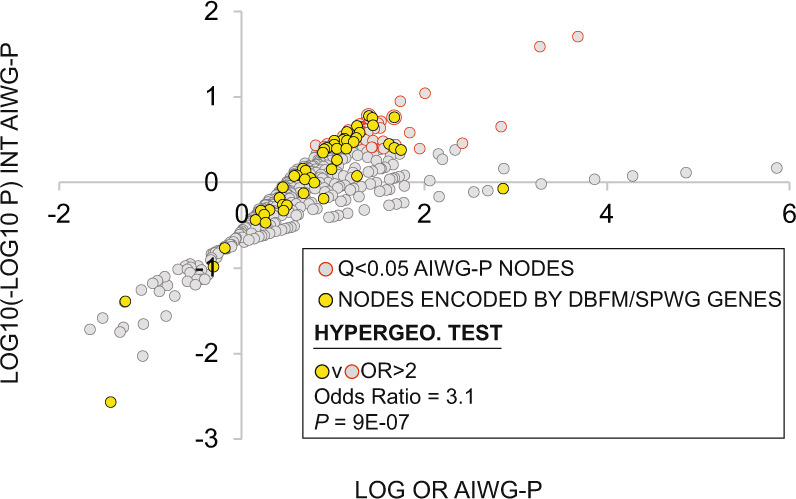
Pathway nodes driving predisposition to AIWG are enriched for nodes required for body fat and postnatal weight gain in mice. In total, 699 pathway nodes included in the AIWG-P HCT intersection analysis (Supplementary file section 4) were plotted as log odds ratio (log OR) against log10(-log10 P). A double-log procedure was required due to the large *p*-value range. Nodes with significant (*q* < 0.05) HCT intersections with the AIWG-P gene set are colored gray with an orange border. Nodes with nonsignificant (*q* > 0.05) intersections with the AIWG-P gene set are colored gray. A hypergeometric test was performed on the overrepresentation in the *q* < 0.05 nodes of nodes encoded by genes in the DBFM/SPWG gene set, indicated in yellow. Refer to the Supplementary file for the specific intersecting nodes (section 4) and the list of annotated DBFM/SPWG genes (section 5). Box acronyms: INT, intersection; OR, odds ratio; *p, p*-value of hypergeometric test.

As candidate transcriptional modulators of AIWG, we next hypothesized that nodes with *q* < 0.05 HCT intersections with the AIWG-P gene set would be enriched for genes whose disruption was associated with underweight or slow weight gain phenotypes in mice. To investigate this further, we assembled a list of genes that (i) were annotated by the Monarch [46], IMPC [47], or MMPC [48] initiatives to the Mammalian Phenotype Ontology phenotypes “decreased total body fat mass (DBFM)” or “slow postnatal weight gain (SPWG)” (Supplementary file section 5). Consistent with their potential participation in signaling pathways mediating AIWG, we found that nodes with significant (*q* < 0.05) HCT intersections with the AIWG-P gene set were robustly enriched (20/59; OR, 3.0, *p* = 9.7e-7) for genes whose disruption is associated with either of the two phenotypes (Fig. 5; Supplementary file section 4, DBFM/SPWG column).

### Cross-species markers of AIWG predisposition are classical antiviral inflammatory effectors with conserved upstream-regulatory nodes

We next wished to determine whether the pathways driving AIWG are conserved between mice and humans. To do this, we compared our blood AIWG-P gene set with a set of 155 genes that were significantly differentially expressed in baseline blood between two groups of subjects segregated on the basis of AIWG-P vs AIWG-R after three months of antipsychotic treatment [20] (Supplementary file section 6). We identified four transcripts in common between the mouse AIWG-P gene set and human AIWG-P FC > 1.25 genes, namely, *Rhd, Ifit1*, *Ifitm3*, and *Rsad2* (OR = 3.2 *p* = 0.04, hypergeometric test; Table 1). *Rhd* encodes the highly immunogenic erythrocyte Rh factor antigen of the Rh blood group system [49]. The three other genes—*Ifit1*, *Ifitm3*, and *Rsad2* (Viperin)—are classic interferon-inducible effectors that oppose DNA and RNA virus infection through a variety of mechanisms, including disruption virus entry [50] and inhibition of viral replication [51–53]. Interestingly, adipose tissue expression of *Rsad2* is increased in obesity and its genetic ablation results in decreased fat mass due to increased thermogenesis [54].

**Table 1:** Candidate biomarkers of AIWG in mouse and human studies.

		FC Pr/Res (*p* < 0.05)	
Symbol	Name	Mouse	Human
*Ifit1*	Interferon Induced Protein With Tetratricopeptide Repeats 1	1.32	1.72
*Ifitm3*	Interferon-induced transmembrane protein 3 (IFITM3)	1.25	1.28
*Rhd*	Rh blood group, D antigen	1.94	1.45
*Rsad2*	Radical S-Adenosyl Methionine Domain Containing 2	1.54	2.56

Four genes (*IFIT1, IFITM3, RHD and RSAD2*) are elevated in AIWG-P individuals relative to AIWG-R individuals.

**Fig. 6:**
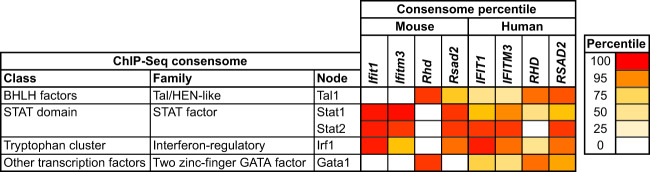
Transcriptional regulatory relationships between cross-species AIWG-P genes and immunomodulatory transcription factors are highly conserved between mice and humans. Shown are percentile rankings of the four cross-species AIWG-P genes in human and mouse ChIP-Seq consensomes for which at least one of both the human and mouse orthologs is a HCT. Refer to the Supplementary file for the human AIWG-P and AIWG-R gene sets (section 6) and the full list of mouse and human percentile rankings for the four genes (section 7).

Given that expression levels of these four transcripts were conserved between mice and humans with respect to predisposition to AIWG-P, we speculated that their upstream signaling nodes would be similarly conserved. Figure 6 compares percentile rankings of the four cross-species’ AIWG-P genes in human and mouse ChIP-Seq consensomes for which at least one of both the human and mouse orthologs is a HCT (the full list of percentile rankings for all four genes is in Supplementary file section 7). This *in silico* analysis also recapitulates previous in vitro studies identifying transcriptional regulatory connections between Stat2 and *Ifit1* [55], Stat1 and *Rsad2* [56], and Tal1, Gata1, and *Rhd* [57]. We observed striking cross-species conservation of the regulatory relationships of the four genes with several of the key immunomodulatory nodes previously identified in the HCT intersection analysis. Moreover, we noted a clear demarcation between the nodes conserved upstream of the canonical interferon-stimulated genes *Ifit1*, *Ifitm3*, and *Rsad2* on the one hand, and *Rhd* on the other. Specifically, nodes upstream of the conserved interferon-stimulated genes comprised members of the STAT and interferon-regulatory families. This was in sharp contrast to the close cross-species-regulatory relationship of *Rhd* with Tal1 and Gata1, which is consistent with the genomic co-occupancy of these factors [58], as well as their fundamental requirement in the development of hematopoietic lineages [36, 37].

### Cross-species markers of AIWG are specific to drug-induced weight gain and are not markers of predisposition to diet-induced weight gain

To determine if the levels of these four genes were specific predictors of drug or diet-induced weight gain, we conducted an additional control study in mice. We analyzed blood-based gene expression of these four genes before exposure to HFD for two weeks (Supplemental Fig. 1A). Mice exposed to HFD for two weeks gained between 2.9 g and 10.8 g each, with the HFWG-P group gaining an average of 9.5 ± 0.86 and HFWG-R group gaining just 4.9 ± 1.34 (Supplemental Fig. 1B). Importantly, there were no significant differences in gene expression of *Ifit1*, *Ifitm3*, Rd, or *Rsad2* in baseline blood samples between these two groups (Supplemental Fig. 1C), suggesting that these genes are specifically markers of drug-induced weight gain and not diet-induced weight gain.

### Conservation of immunomodulatory transcriptional networks predisposing to AIWG-P in mice and to weight gain and elevated body mass index in humans

**Fig. 7:**
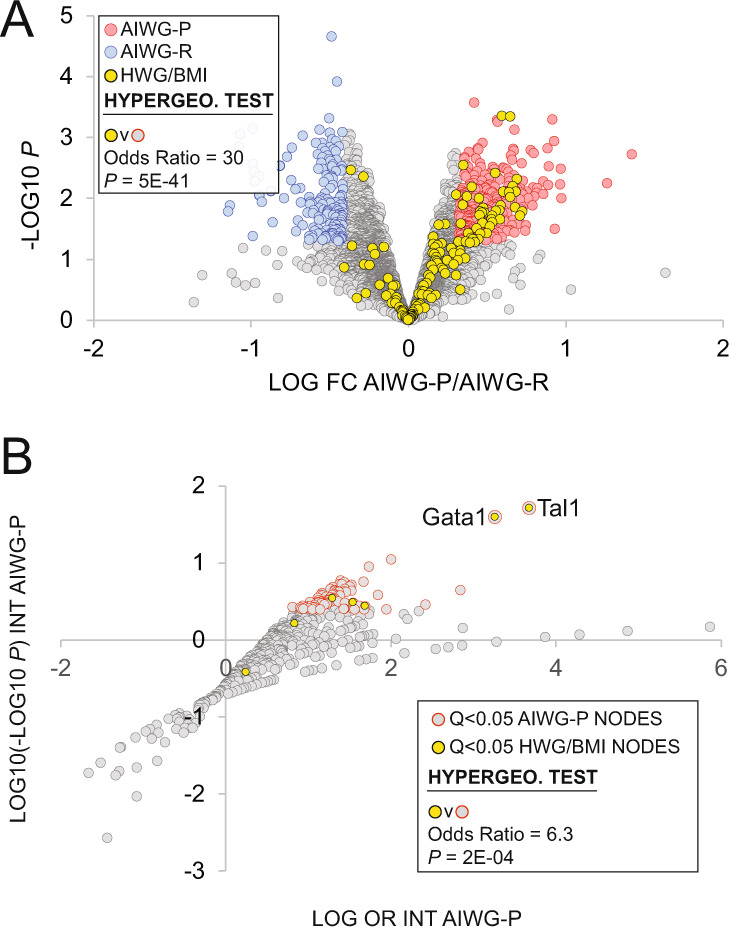
Conservation of immunomodulatory pathways supporting AIWG in mice and weight gain and elevated body mass index in humans. **A**. Volcano plot of differential gene expression between AIWG-P and AIWG-resistant mice. The AIWG-P gene set (AIWG-P/AIWG-R FC > 1.25, *p* < 0.05) is indicated in red and the AIWG-R gene set (AIWG-P/AIWG-R FC < 0.75, *p* < 0.05) is indicated in blue. *p* > 0.05 genes are colored gray. A hypergeometric test was performed on the overrepresentation in the AIWG-P gene set of mouse orthologs of the human HWG/BMI gene set, indicated in yellow. Refer to the Supplementary file for the specific intersecting genes (section 2, Mm HWG/BMI column) and the full human HWG/BMI gene set (section 8). Box acronyms: INT, intersection; OR, odds ratio; *p, p*-value of hypergeometric test. **B** In total, 699 pathway nodes included in the AIWG-P HCT intersection analysis (Supplementary file section 4) were plotted as log odds ratio (log OR) against log10(-log10 P). A double-log procedure was required due to the large *p*-value range. Nodes with significant (*q* < 0.05) HCT intersections with the AIWG-P gene set are colored gray with an orange border. Nodes with nonsignificant (*q* > 0.05) intersections with the AIWG-P gene set are colored gray. A hypergeometric test was performed on the overrepresentation in the *q* < 0.05 nodes of mouse orthologs of nodes with significant (*q* < 0.05) HCT intersections with the human HWG/BMI gene set, indicated in yellow. Refer to the Supplementary file for the specific intersecting nodes (section 4), the full HWG/BMI gene set (section 8), and the full results of the human HCT intersection analysis of the HWG/BMI gene set (section 9). Box acronyms: INT, intersection; OR, odds ratio; *p, p*-value of hypergeometric test.

The conservation of AIWG-P genes and their transcriptional regulators in the context of the mouse and human AIWG studies led us to consider whether the AIWG-P transcriptional regulatory network might be more broadly conserved in whole-body fat metabolism in humans. To investigate this further we retrieved a set of 157 human genes whose expression levels in blood were shown to have a strong positive correlation with weight and body mass index (BMI) in humans (designated HWG/BMI genes; Supplementary file section 8; [59]). Consistent with the connection between the AIWG-P gene set and downstream inflammatory processes (Fig. 3), pathway analysis of this gene set had indicated enrichment of inflammatory genes [59]. When we overlaid mouse orthologs of the HWG/BMI genes on the AIWG-P and AIWG-R gene sets, the high degree of overlap of mouse orthologs of the HWG/BMI genes with the AIWG-P gene set (40/157; OR = 30, *p* = 5e-41) was in striking contrast to the complete absence of overlap with the AIWG-R gene set (0/169; *p* = 1) (Fig. 7A; Supplementary file section 2, column “Mm HWG/BMI”). As shown in Table 2, genes common to the AIWG-P and HWG/BMI gene sets map to a broad range of functional categories, classes, and families annotated according to SPP convention as previously described. Of particular note, given known connections between the ubiquitin–proteasome system, innate immunity and obesity [60–62] is the substantial overrepresentation (6/40; OR = 11.4, *p* = 1e-5, hypergeometric test) of genes encoding enzymes in the E2 ubiquitin-conjugating and E3 ubiquitin ligase classes.

**Table 2:** Functional categorization of genes in common between the mouse AIWG-P gene set and the human HWG/BMI gene set.

Category	Class	Family	Symbol
Enzymes	Dehydratases	Carbonic anhydrases (CA)	*Car2*
	E2 ubiquitin conjugating enzymes	Ubiquitin conjugating enzymes E2 (UBE2)	*Ube2f*
			*Ube2h*
	E3 ubiquitin ligases	Makorin (MKRN)	*Mkrn1*
		Membrane associated ring-CH-type finger	*Marchf8*
		Tripartite motif-containing (TRIM)	*Trim10*
			*Trim58*
	Kinases	Membrane palmitoylated proteins	*Mpp1*
	Lyases	Ferrochelatases	*Fech*
	Oxidases	Spermine oxidases	*Smox*
	Peptidases	Cathepsins (CTS)	*Ctsb*
	Peroxidases	Peroxiredoxins (PRDX)	*Prdx2*
	Regulatory factors	Protein phosphatase 1 regulatory subunits	*Bcl2l1*
	Transferases	Hydroxymethylbilane synthases	*Hmbs*
	Other enzymes	Glutamate-ammonia ligases	*Glul*
		Glutaredoxin	*Glrx5*
Transcription factors	E2F/FOX	E2F	*E2f2*
Co-nodes	Cell cycle, cell division & DNA repair	Cell cycle checkpoint proteins (RAD)	*Rad23a*
		Growth arrest and DNA damage inducible	*Gadd45a*
	CNS Proteins	Synuclein	*Snca*
	Cytoskeleton components & regulators	Dematin actin binding protein	*Dmtn*
		Tropomyosin	*Tpm1*
	F box domain	F-box protein	*Fbxo7*
			*Fbxo9*
	Family with sequence similarity	Family with sequence similarity member	*Fam104a*
			*Fam210b*
	Globins	Hemoglobin subunit theta	*Hbq1b*
	Membrane proteins	Transmembrane and coiled-coil domain	*Tmcc2*
	Receptor associated factors	GABA type A receptor associated protein like	*Gabarapl2*
		Glutamate ionotropic receptor NMDA type subunit associated	*Grina*
	Ring finger proteins	Ring finger protein	*Rnf10*
			*Rnf11*
	Stress response factors	DnaJ heat shock protein (Hsp40) member	*Dnaja4*
	Transporters & transport proteins	Solute carrier superfamily member	*Slc25a37*
			*Slc48a1*
			*Slc4a1*
	Other co-nodes	BCL2 interacting protein like	*Bnip3l*
		Cellular repressor of E1A stimulated genes	*Creg1*
		FUN14 domain containing	*Fundc2*
		PITH domain containing	*Pithd1*

To evaluate whether this strong identity between mice and humans extended to upstream transcriptional drivers of these gene sets, we next identified nine human nodes with significant (*q* < 0.05) HCT intersections with the HWG/BMI gene set (Supplementary file section 9). The gene encoding the top-ranked node in this analysis, *TAL1*, has been identified as a human BMI-associated GWAS locus, and knockout of the *Drosophila* ortholog results in decreased percent body fat [63]. Of these nine nodes, mouse orthologs for seven had been included in the earlier mouse HCT intersection analysis (Supplementary file section 4, column *q* < 0.05 INT HWG/BMI). Reflecting a strong conservation between the AIWG-P and HWG/BMI gene sets at the transcriptional regulatory level, mouse orthologs of five of these seven nodes (Tal1, Gata1, Zmiz1, Cebpd, and Smad1; OR 6.3, *p* = 2e-4, hypergeometric test) had *q* < 0.05 intersections with the mouse AIWG-P gene set (Fig. 7B). Notably, of these five nodes, all but Zmiz1 have well documented roles in the inflammatory and immunomodulatory signaling. Moreover, Tal1/TAL1 and Gata1/GATA1 were the top-ranked nodes in both analyses (Fig. 7B). Collectively, these data indicate that inflammatory and immunomodulatory signaling pathways contributing to weight gain and BMI are strongly conserved between mice and humans at the levels of both transcriptional drivers (pathway nodes) and effectors (pathway node genomic targets).

## Discussion

In this study, we found that immunomodulatory transcriptional effectors and their upstream regulatory drivers strongly predispose mice to AIWG. In validation of our findings, we showed that genes encoding these upstream regulatory drivers are enriched for genes whose disruption in mice is associated with reduced body fat mass and slow postnatal weight gain. We proceeded to identify a minimal, cross-species AIWG-P signature that has the potential to determine the likelihood of AIWG in individual patients. We then demonstrated robust conservation between AIWG-P effectors and transcriptional drivers and those associated with weight gain and body mass index in humans. Collectively, our results provide mechanistic insight into the known links between inflammation, immunomodulation, obesity, and the metabolic syndrome [33, 34], as well as the extent to which pathways driving these connections are conserved between mice and humans.

Combining conventional pathway analysis of AIWG-P genes with a dissection of their regulatory relationships with upstream signaling pathway nodes, our analysis affords insights into the mechanisms underlying AIWG that are unavailable through pathway analysis alone. Initial GO pathway analysis of the underlying biological functions of AIWG-P genes indicated a strong link with inflammatory processes, which are known drivers of the development of metabolic disease [33, 34]. This was corroborated and extended by the HCT intersection analysis, which illuminated robust conservation of the regulatory relationships of AIWG-P genes with numerous inflammatory nodes in both mice (Fig. 4) and humans (Fig. 6), suggesting that individuals with a primed immune system are highly susceptible to AIWG. Significantly, while genes encoding AIWG-P nodes were enriched for genes whose disruption is associated with reduced body fat mass and slow postnatal weight gain (Fig. 5), no such enrichment was observed for the AIWG-P gene set itself (data not shown). These results indicate that transcriptional drivers of AIWG-P, rather than transcriptional effectors, are more likely to have nonredundant roles in the regulation of AIWG.

Although inflammation and immunomodulation were the primary thematic connections across AIWG-P genes, other paradigms were evident. For example, the significant intersections of the AIWG-P gene set with HCTs of several transcription factors with characterized roles in circadian biology were of additional interest in light of our recent studies showing that the circadian rhythm plays an important role in AIWG [41]. This suggests that variation in expression of genes that regulate the circadian rhythm also contributes to the risk potential of AIWG. Interestingly, the AIWG-P HCT intersection for the adipogenic master regulator Pparg is relatively small (4.6e-2), suggesting either that members of this gene set are indirect Pparg targets, or that they represent a module of transcriptional effectors that predispose to weight gain via a mechanism that is largely independent of classical adipogenic pathways. In addition, although the primary motivation of this study was the characterization of markers of AIWG and their transcriptional drivers, this analysis also paves the way for future studies into the role of AIWG-P nodes with no previously reported connections to whole-body energy metabolism, including Anp32e (*q* = 9e-3) Six4 and Zfp281 (both *q* = 8e-3).

In this study, we focused on female mice as they most closely model the antipsychotic-induced weight gain observed in human patients. Notably, there is a growing body of literature that women are more susceptible to AP-induced weight gain and metabolic side effects than men [64–67]. In addition, olanzapine was dosed in the diet (54 mg/kg diet) resulting in mouse plasma levels (21 ± 5 ng/mL) that are similar to the levels observed in humans treated with olanzapine (10–50 ng/mL) [17]. In previous dosing studies in mice, olanzapine dosing above 25–100 mg/kg in the diet had equivalent effects on body weight gain [17]. Importantly, the terminal elimination half-life of OLZ in mice is approximately 3 hr [68] compared with 21–54 h in humans [69], depending on a range of factors including sex and ethnicity [70]. Therefore, to ensure exposure across the course of the day and night, we opted for diet administration, whereby self-administration ensures exposure in rodents across the day.

In summary, we have identified transcriptional regulatory networks comprising immunomodulatory signaling nodes and their downstream effectors that predispose to AIWG. In addition, we identified a group of cross-species AIWG-P effectors whose regulatory relationships with several immunomodulatory nodes are highly conserved between mice and humans. To further validate these cross-species effectors as biomarkers in predicting AIWG, future studies will be needed involving independent replicate groups of mice and humans. In validation of our study, we found that numerous AIWG-P nodes are encoded by mouse genes with known adipose phenotypes, and that the immunomodulatory AIWG-P transcriptional networks are conserved in predisposing to weight gain and elevated body mass index in humans. These points of convergence underscore the relevance of immunomodulatory transcriptional networks identified in this study to AIWG and metabolic disease more generally across mice and humans.

## Supplementary information

supplemental file legends

supplemental figure

supplemental tables
