# Supplementary material for: Conserved immunomodulatory transcriptional networks underlie antipsychotic-induced weight gain
Source: Transl Psychiatry. 2021 Jul 22;11:405. doi: 10.1038/s41398-021-01528-y (PMC8296828; doi:10.1038/s41398-021-01528-y)
Supplement: Supplementary file 1 — supplemental file legends [file 41398_2021_1528_MOESM1_ESM.docx]

**Supplementary file section legends**

Supplementary **figure 1. Cross-species markers of AIWG are specific to drug-induced weight gain and are not markers of predisposition to diet-induced weight gain.** A. Mice exposed to HFD for 2 weeks gained between 2.9 g and 10.8 g each, with the HFWG-P (prone) group gaining an average of 9.5 ± 0.86 and the HFWG-R (resistant) group gaining just 4.9 ± 1.34 (B). C. Importantly, there was no significant differences in gene expression of *Ifit1*, *Ifitm3*, Rd or *Rsad2* in baseline blood samples between these two groups suggesting these genes are specifically markers of drug-induced weight gain and not diet-induced weight gain. * = *p* <0.05 determined by students t-test.

Supplementary **file section 1.** Primer sequences for q-PCR

Supplementary **file section 2.** Mouse AIWG-P and AIWG-R gene sets. 558 genes were significantly differentially expressed (FC > ± 1.25, *p* < 0.05) at baseline between the weight gain ‘prone’ (AIWG-P) and ‘resistant’ (AIWG-R) groups of mice of which 389 were elevated in AIWG-P > AIWG-R (AIWG-P gene set), and 169 were elevated in AIWG-R > AIWG-P (AIWG-R gene set). Column “Mm HWG/BMI” indicates mouse orthologs of human genes in section 7.

Supplementary **file section 3.** PANTHER Gene Ontology analysis of the mouse AIWG-P gene set (q < 0.05) No significant (*q* < 0.05) process enrichments were observed in the AIWG-R gene set.

Supplementary **file section 4.** ChIP-Seq high confidence transcriptional target (HCT) intersection analysis of AIWG-P and AIWG-R gene sets. The “DBFM/SPNWG” column indicates analyzed nodes encoded by genes in section 4. ‘q < 0.05 INT HWG/BMI” column indicates mouse orthologs of human nodes with significant (*q* < 0.05) HCT intersections with the HWG/BMI gene set (see section 7). INT: intersection; OR: odds ratio; *P,* p-value; *Q*, FDR-corrected *p*-value.

Supplementary **file section 5**. DBFM/SPWG gene set. This is a set of genes mapped to Mammalian Phenotype Ontology terms “decreased body fat mass” (DBFM; MP: 0014143) and “slow postnatal weight gain” (SPWG; MP:0008489) by Monarch, MMPC and IMPC.

Supplementary **file section 6.** Human AIWG-P and AIWG-R gene sets: 155 human genes with significant differential expression between weight gain and no weight gain groups before drug treatment. (20)

Supplementary **file section 7.** Mouse and human 5 kb ChIP-Seq node consensome percentile rankings of *Ifit1*, *Ifitm3*, *Rhd* and *Rsad2*.

Supplementary **file section 8.** HWG/BMI gene set: "black" module genes exhibiting strong positive correlation between blood expression levels and weight or BMI in humans (Joseph et al. (2019) Sci Rep 9, 7447. https://doi.org/10.1038/s41598-019-43881-5

Supplementary **file section 9.** ChIP-Seq high confidence transcriptional target (HCT) intersection analysis of the HWG/BMI gene set. The universe for the intersection was set at the total number of coding sequences represented in the Affymetrix U133A 2.0 Array, which was the transcriptomic platform used in the human weight gain / body mass index study (Joseph et al. (2019) Sci Rep 9, 7447. https://doi.org/10.1038/s41598-019-43881-5). INT: intersection; OR: odds ratio; *P,* p-value; *Q*, FDR-corrected *p*-value.
