# Supplementary figures and images for: Conserved immunomodulatory transcriptional networks underlie antipsychotic-induced weight gain
Source: Transl Psychiatry. 2021 Jul 22;11:405. doi: 10.1038/s41398-021-01528-y (PMC8296828; doi:10.1038/s41398-021-01528-y)

**A**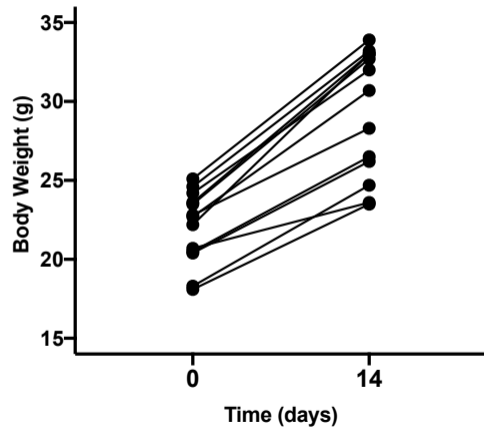**B**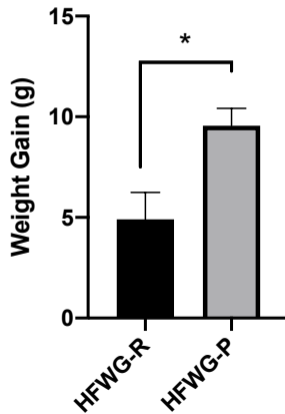**C**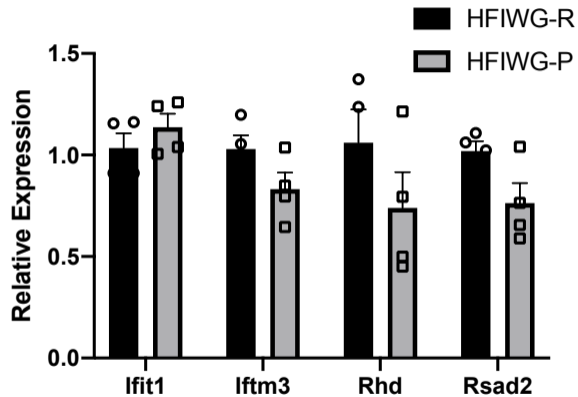

Supplement: Supplementary file 2 — supplemental figure [file 41398_2021_1528_MOESM2_ESM.pdf]
